# Glioma Recurrence following Surgery: Peritumoral or Perilesional?

**DOI:** 10.3389/fneur.2016.00052

**Published:** 2016-03-31

**Authors:** Boudewijn van der Sanden, David Ratel, François Berger, Didier Wion

**Affiliations:** ^1^INSERM UA01, Clinatec, Institut E.J. Safra, CEA, Grenoble, France; ^2^Clinatec, Institut E.J. Safra, CEA, Grenoble, France

**Keywords:** glioma, surgery injury, microenvironment, inflammation, recurrence, paradigm shift

## Background

In comparison to other cancers, gliomas are locally invasive cancers that seldom metastasize. Glioma cells infiltrate brain parenchyma up to several centimeters from the tumor core. Therefore, their recurrence occurs at the margin of tumor resection in about 90% of patients (1). Significant effort is put into analyzing peritumoral regions to understand the mechanisms of glioma recurrence after surgery (1–4). Such studies are done on peritumoral biopsies collected either before or during surgery. This comment seems incidental but is critical.

## Surgical Brain Injury Reshapes the Peritumoral Landscape

A critical overlooked point in neurooncology is that brain surgery triggers a wound–healing–inflammatory process in the resection margins where residual glioma cells are. Glioma resection is lesional, and it generates dramatic rearrangements in the peritumoral brain region. The protumorigenic role of wounds and the vicious connection between inflammation and cancer have long been established in other tissues (5, 6). Regarding brain tissue, we already know that in the minutes following cortical and parenchymal incisions, surgical brain injury (SBI) induces (i) a burst of glutamate and ATP, (ii) the release of mutagenic reactive oxygen species, and (iii) an influx of blood clotting factors and serum components (7, 8). Fibroblasts, mesenchymal stem cells, and other blood-borne cells, including inflammatory cells, also invade the resection margin (7, 8). This in turn leads to a further release of cytokines and growth factors. Small ischemic areas around the resection cavity, which are an important driver for malignant progression, are also observed (9, 10). Hallmarks of this perilesional region include inflammation, angiogenesis, glial cell proliferation, reactive gliosis, and fibrosis (7, 8). Indeed, SBI reshapes the peritumoral landscape into a protumorigenic environment.

## Conclusion

Peritumoral biopsies collected before or during surgery are not representative of the SBI-induced protumorigenic microenvironment. Hence, critical information is missing to understand and prevent tumor recurrence. Personalized medicine and targeted therapies must integrate new technologies to improve our understanding of the SBI zone. Considering the resection margins as peritumoral or as perilesional is more than a simple matter of vocabulary. It is a necessary paradigm shift to understand and treat glioma recurrence after surgery.

## Author Contributions

All authors listed, have made substantial, direct, and intellectual contribution to the work, and approved it for publication.

## Conflict of Interest Statement

The authors declare that the research was conducted in the absence of any commercial or financial relationships that could be construed as a potential conflict of interest.
